# Elemental Concentrations in the Seed of Mutants and Natural Variants of *Arabidopsis thaliana* Grown under Varying Soil Conditions

**DOI:** 10.1371/journal.pone.0063014

**Published:** 2013-05-06

**Authors:** Stephen C. McDowell, Garo Akmakjian, Chris Sladek, David Mendoza-Cozatl, Joe B. Morrissey, Nick Saini, Ron Mittler, Ivan Baxter, David E. Salt, John M. Ward, Julian I. Schroeder, Mary Lou Guerinot, Jeffrey F. Harper

**Affiliations:** 1 Department of Biochemistry and Molecular Biology, University of Nevada, Reno, Nevada, United States of America; 2 Division of Biological Sciences, University of California, San Diego, California, United States of America; 3 Department of Biological Sciences, Dartmouth College, Hanover, New Hampshire, United States of America; 4 Institute of Biological and Environmental Science, University of Aberdeen, Aberdeen, Scotland, United Kingdom; 5 Department of Plant Biology, University of Minnesota, St. Paul, Minnesota, United States of America; 6 Department of Biological Sciences, University of North Texas, Denton, Texas, United States of America; 7 United States Department of Agriculture–Agricultural Research Service, Plant Genetics Research Unit, Donald Danforth Plant Science Center, St. Louis, Missouri, United States of America; Ghent University, Belgium

## Abstract

The concentrations of mineral nutrients in seeds are critical to both the life cycle of plants as well as human nutrition. These concentrations are strongly influenced by soil conditions, as shown here by quantifying the concentration of 14 elements in seeds from *Arabidopsis thaliana* plants grown under four different soil conditions: standard, or modified with NaCl, heavy metals, or alkali. Each of the modified soils resulted in a unique change to the seed ionome (the mineral nutrient content of the seeds). To help identify the genetic networks regulating the seed ionome, changes in elemental concentrations were evaluated using mutants corresponding to 760 genes as well as 10 naturally occurring accessions. The frequency of ionomic phenotypes supports an estimate that as much as 11% of the *A. thaliana* genome encodes proteins of functional relevance to ion homeostasis in seeds. A subset of mutants were analyzed with two independent alleles, providing five examples of genes important for regulation of the seed ionome: *SOS2*, *ABH1*, *CCC*, *At3g14280* and *CNGC2*. In a comparison of nine different accessions to a Col-0 reference, eight accessions were observed to have reproducible differences in elemental concentrations, seven of which were dependent on specific soil conditions. These results indicate that the *A. thaliana* seed ionome is distinct from the vegetative ionome, and that elemental analysis is a sensitive approach to identify genes controlling ion homeostasis, including those that regulate gene expression, phospho-regulation, and ion transport.

## Introduction

Ion homeostasis is a complex process that is essential to the health and survival of all organisms. While at least 14 mineral nutrients are essential for higher plants [1], they can also be toxic if present in excess. Most land plants obtain their mineral nutrients from the soil, with homeostasis involving the regulation of uptake, binding, transport and sequestration; each of which is controlled by a network of genes. The resulting elemental composition of a specific tissue or cell has been referred to as the ionome [2]–[4].

Large scale screens to identify ionomic mutants have been undertaken in yeast, *A. thaliana* and Lotus [5]–[8]. These studies have provided estimates that approximately 5% of the genome might be involved in ion homeostasis. Genetic variation controlling the leaf ionome has also been observed between naturally occurring populations of *A. thaliana* [9]–[17] and used in several cases to identify genes of phenotypic consequence [18]–[23]. Ionomic techniques have also been applied to crop plants including maize, rice, tomato and wheat [24]–[27].

Land plants have evolved the ability to adapt to highly variable soil conditions. Many studies have correlated different soil conditions with changes in elemental concentrations [14], [17], [28]–[30]. Soils that are deficient in specific nutrients, such as Fe and P, can sometimes be diagnosed by assaying the leaf ionome for a specific pattern of changes [31]. However, relatively little is known about the estimated 5% of the genome expected to regulate ion homeostasis, and even less about how genetic networks coordinate plant-environment interactions.

The purpose of this study was to explore different strategies to identify genes regulating ion homeostasis in seeds. While previous screens have uncovered mutations affecting the leaf ionome, this study was focused on the seed for two reasons. First, the elemental concentrations of seed are expected to be strongly influenced by the physiological functions of the root and shoot; thereby providing a test that can potentially detect ionome defects throughout the plant [3]. Second, seed represent a primary source of mineral nutrition in the human diet, as well as a gateway for the entry of toxic elements into the food chain, and therefore an important target for biofortification or toxic element exclusion.

Here we report on a pilot study in which we quantified 14 elements in the seed of plants grown under four different soil conditions: standard, or modified with NaCl, heavy metals, or alkali. This survey included mutants corresponding to 760 genes, including membrane proteins, transcription factors, stress regulated genes, and unknowns. In addition, we included a comparison of 10 natural variants of *A. thaliana*. Our survey provides evidence that as much as 11% of the *A. thaliana* genome encodes proteins of functional relevance to the seed ionome. Here we identify seed ionome phenotypes for mutations in 15 different genes, five of which were corroborated with two independent mutant alleles. Phenotypes were also observed in eight of the nine accessions, in comparison to Col-0. The use of four different soil conditions revealed several conditional phenotypes, providing evidence that the seed ionome is highly sensitive to changes in the soil environment.

## Results

### Soil Modifications Affect Ion Homeostasis in Col-0 Seed

**Figure 1:**
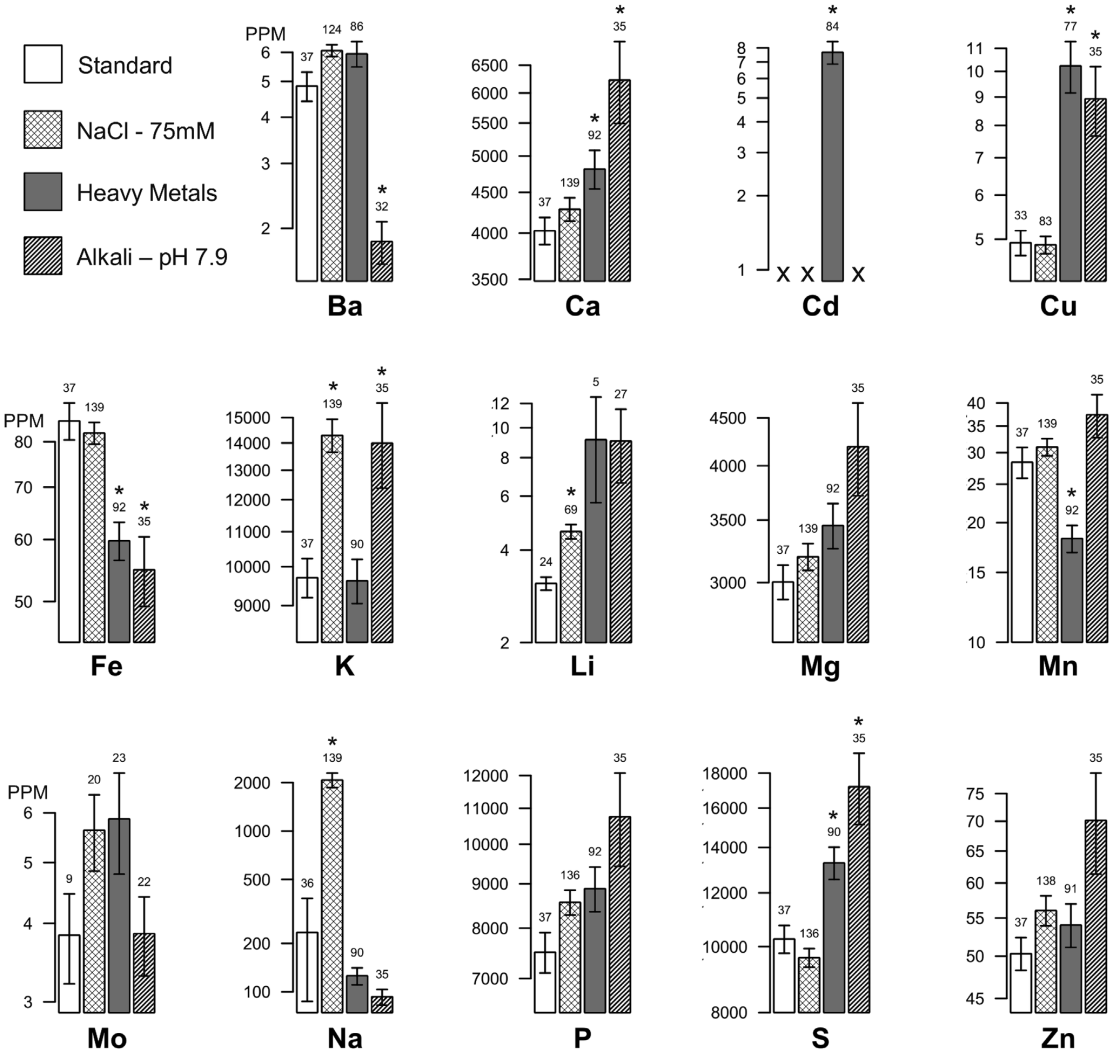
The elemental concentrations of Col-0 seed change under modified soil conditions. The average (±SE) concentration of each element is given in PPM and plotted on the y-axis on a log scale. In total, 139 flats were processed for NaCl soil, 92 for heavy metal soil, 35 for alkali soil and 37 for untreated soil. However, some elements were not detected in every flat. The numbers above the columns represent the number of flats from which data was collected (i.e., n-values). Circumstances for which no data was collected are represented by an “X”. ^*^ Elements for which a statistically significant difference between standard and modified soil conditions was observed (t-test, p<0.05, Bonferroni multiple test correction).

To evaluate the impact of growth conditions on the *A. thaliana* seed ionome, the concentration of 14 elements (Ba, Ca, Cd, Cu, Fe, K Li, Mg, Mn, Mo, Na, P, S and Zn) was quantified in the seed of plants grown under four different soil conditions: standard, or modified with NaCl (75 mM NaCl), heavy metals (Al, As, Cd, Co, Ba, Cs, Cr, Cu, Ca, Fe, Mn, Mg, Li, Rb), or alkali (pH ∼ 7.9 with CaO). Under standard conditions, the average elemental concentrations of Col-0 seed were comparable to previous reports [14]. Under heavy-metal growth conditions, most of the elements added to the soil accumulated in the seeds at concentrations below the lower limit of detection due to sensitivity limitations in the ICP-AES instrument used in this study. Each soil modification was chosen to challenge plants with a sub-optimal soil environment, while still allowing plants to grow and provide sufficient seed for ICP analyses. For example, Col-0 plants showed no consistent differences in plant health, bolt length, or seed set between the standard and 75 mM NaCl soil conditions. Furthermore, a quantitative analysis of Col-0 seed size found no statistically significant differences between standard and 75 mM NaCl soil conditions (Figure S1). Nevertheless, each of the three challenge conditions resulted in a unique perturbation of the Col-0 seed ionome (Figure 1). NaCl conditions increased relative concentrations of K, Li and Na; heavy metal conditions increased Ca, Cd, Cu, and S, but decreased Fe and Mn; and alkali conditions increased Ca, Cu, K, and S, but decreased Ba and Fe. This indicated that the chosen conditions were sufficient to alter ion homeostasis in the seed, supporting an expectation that these conditions could be used in the context of a mutant screen to increase the probability of identifying mutations that alter ion accumulation in the seed.

**Figure 2:**
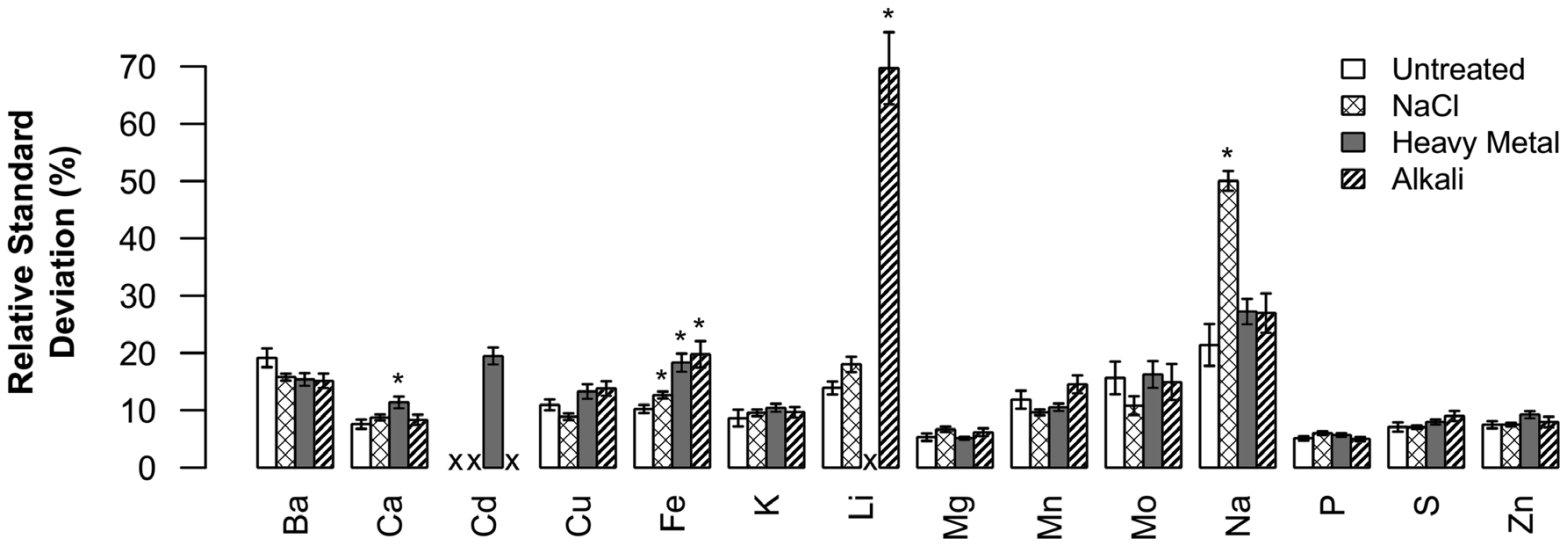
Similar variability was found for 9 of the 14 elements under all soil conditions. Columns represent the average variability (±SE) of an element in Col-0 control seeds under a specific soil condition, expressed in terms of the standard deviation relative to the mean (%RSD, percent relative standard deviation). In total, 139 flats were processed for NaCl soil, 92 for heavy metal soil, 35 for alkali soil and 37 for untreated soil. However, some elements were not detected in every flat. The exact n-value for each circumstance can be found in Figure 1. Circumstances for which no data was collected are represented by an “X”. ^*^ Elements for which a statistically significant difference between standard and modified soil conditions was observed (t-test, p<0.05, Bonferroni multiple test correction).

Plants were grown in trays of 32, with eight Col-0 control plants present in each tray. An experimental plant was considered to have a statistically significant change in elemental concentrations (i.e., an ionome phenotype) if one or more elements was different from the Col-0 control mean by three or more standard deviations (3 s.d.). Therefore, the sensitivity of phenotype detection was directly related to the variability of each specific element within the Col-0 control seeds. Figure 2 shows the variability of each element in Col-0 control seeds under each soil condition, expressed in terms of the standard deviation relative to the mean (%RSD, percent relative standard deviation). While each tray was considered individually, the average variability of eight elements across the entire dataset (Ca, Cu, K, Mg, Mn, P, S, and Zn) was between 5–15%RSD for every soil condition. Higher variability (15–30%RSD) was observed for Ba, Cd, Li, Fe, Mo, and Na, resulting in lower sensitivity of phenotype detection. The highest variation was seen for Na (50%RSD) and Li (70%RSD), specifically in soils modified with NaCl and alkali, respectively.

### Mutant Screen: T-DNA Insertion and Fast Neutron Deletion Lines

To identify genes that contribute to the regulation of the seed ionome, the elemental concentrations of mutant and Col-0 (i.e., wild-type) control seeds were compared under different soil conditions (File S1). Mutant lines representing 760 genes were chosen for analysis and included a nearly proportional representation from all gene ontology (GO) functional categories (Figure S2). The exception was transport-related genes, which were over-represented in our screen by approximately three-fold. The best candidates for disruption of gene functions were chosen when selecting mutant alleles for analysis. For example, all mutants used had T-DNA insertions located between the start and stop codons of the target gene. However, many of the lines had insertions located in introns, and no analyses were done to confirm the absence of corresponding full-length mRNA expression. In addition, most of the T-DNA insertion lines obtained from the stock center as homozygous mutants were used without re-confirming their zygosity. Thus, it is possible that some mutant phenotypes were missed in this survey due to leaky expression or mis-represented zygosity.

Three different labs screened subsets of mutants for the 760 genes, each using a different soil modification. The results were compiled for this report (File S1). 731 genes were screened in NaCl soil, 203 genes in heavy metal soil, 88 genes in alkali soil and 38 genes in untreated soil. Mutant lines were initially subjected to a first pass screen in which at least two plants were analyzed. The first pass screen identified 299 genes as candidates for having ionomic phenotypes; revealing potential increases and decreases in every element detectable with our instrumentation. A follow up analysis of genes randomly selected from the 299 candidates yielded a 22% confirmation rate (File S1), suggesting that ∼9% of the 760 total genes analyzed in this study are likely to have ionomic phenotypes. An extension of this allows us to speculate that 7%–11% (at 95% confidence) of the *A. thaliana* genome encodes proteins of functional relevance to the seed ionome.

**Figure 3:**
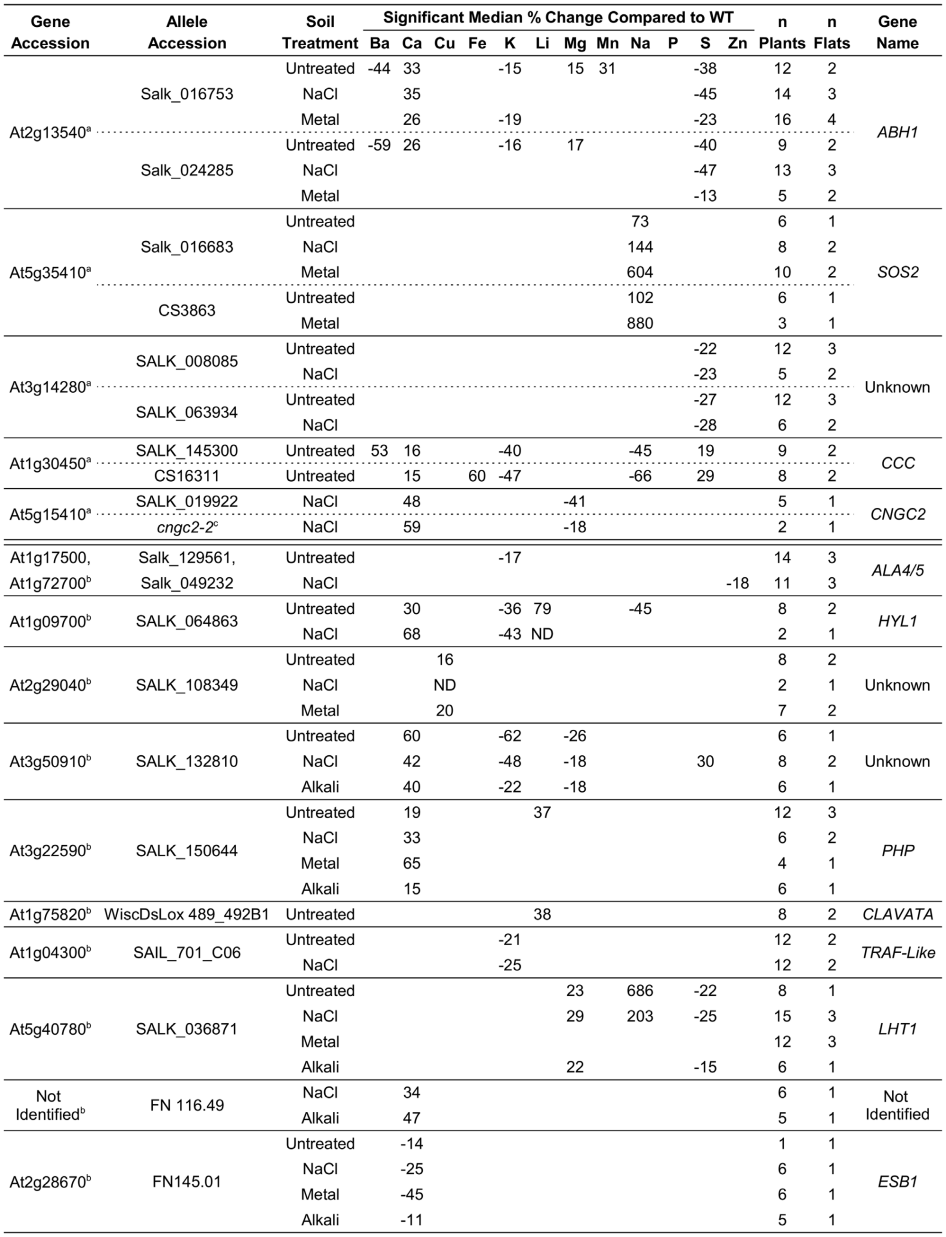
Percent changes in mutant elemental concentrations identify 15 genes that regulate the seed ionome. Data represent the median percent changes in the concentration of elements with reproducibly significant differences between the mutants and the Col-0 wild-type controls. An ionomic change was considered significant if more than three standard deviations from the control mean and reproducible if consistent across multiple flats, soil conditions, or alleles. Circumstances for which no data was collected are represented by “ND”. ^a^ Genes with ionomic phenotypes corroborated by two independent alleles ^b^ Candidate genes with putative ionomic phenotypes evaluated with only one allele ^c^ T-DNA insertional mutant of CNGC2 [66]: ig# 581 and Harper Lab ss57.

Ionomic phenotypes were corroborated in mutant lines corresponding to 15 genes (Figure 3). The corroboration included meeting stringency criteria such as: reproducibility across multiple flats, soil conditions, or multiple alleles. Of these 15 genes, five were shown to have mutant phenotypes supported by two independent alleles (*ABH1, SOS2, At3g14280, CCC, and CNGC2*). Corroboration with two alleles provides strong evidence that the ionomic phenotype is associated with a specific gene, as opposed to a possible second site mutation in some other gene. However, ten of the 15 genes with ionomic phenotypes were only analyzed with a single allele (Figure 3), and still need to be verified by second alleles or transgene rescue experiments.

### Accession Screen

**Figure 4:**
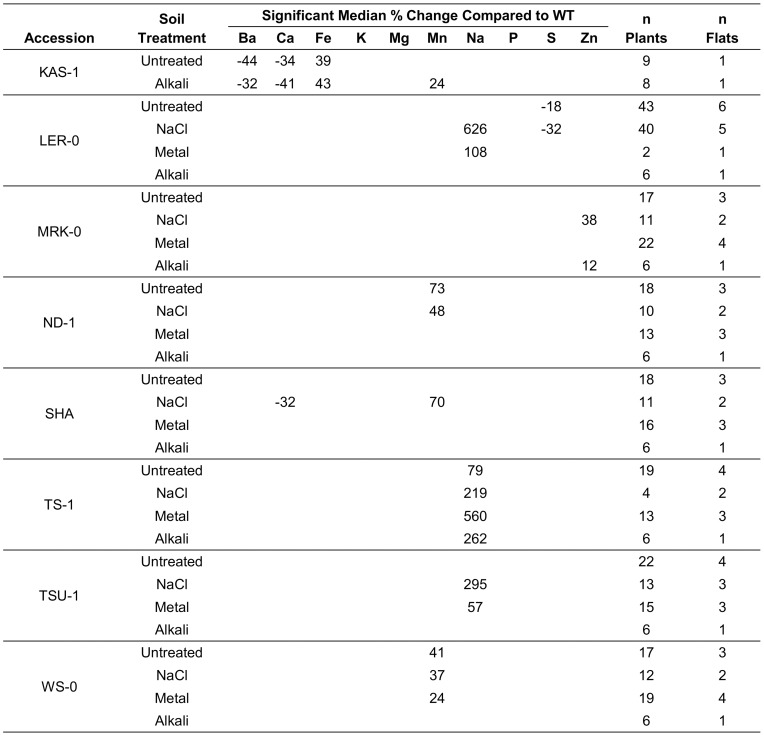
Percent changes in elemental concentrations relative to Col-0 reveal soil-dependent differences in natural variants. Data represents the median percent change in the concentration of elements with reproducibly significant differences between the experimental accessions and the Col-0 control accession. An ionomic change was considered significant if more than three standard deviations from the control mean and reproducible if consistent across multiple flats, soil conditions, or alleles.

To explore the extent of elemental variation between naturally occurring populations of *A. thaliana*, 10 accessions (Col-0, Cvi-1, Kas-1, Ler-0, Mrk-0, Nd-1, Sha, Ts-1, Tsu-1 and WS-0) were grown under standard, NaCl, heavy metal and alkali soil conditions. Compared to Col-0, the only accession that did not show an ionomic difference under one or more soil conditions was Cvi-1. In contrast, the other eight accessions (Kas-1, Ler-0, Mrk-0, Nd-1, Sha, Ts-1, Tsu-1 and WS) showed a total of 13 ionomic changes (Figure 4). For example, Kas-1 showed an increase in Fe, while Mrk-0 showed an increase in Zn. In the example of a Zn increase in Mrk-0, the increase was soil-dependent, as it was seen here in seed from plants grown under a NaCl challenge, but not with unmodified soil.

## Discussion

To identify genes regulating ion homeostasis in seeds, this survey utilized four different soil conditions as a way to challenge plants to adjust their ion homeostasis set points, and thereby help reveal conditional phenotypes. Fourteen elements were quantified in an analysis of mutants corresponding to 760 genes, as well as the natural variation present in nine different accessions compared to Col-0.

### Soil Modifications Alter Seed Ion Homeostasis

Two lines of evidence support a conclusion that the challenge conditions used in our assays altered ion homeostasis in *A. thaliana* seed. First, each challenge uniquely altered the concentration of elements in the seed of Col-0 control plants (Figure 1). The fact that changes were seen in elements other than those used to modify the soil supports an expectation that homeostasis is maintained by interconnected regulatory networks, and that each of the modified soil conditions used here was sufficient to perturb some of these connections. Second, in both the mutant and accession screens (Figures 3 and 4) many of the phenotypes were observed to be soil-specific. For example, while five elements showed significant changes in *abh1* seed obtained from plants grown under standard soil conditions, the only change that was consistent under all four soil conditions was a decrease in sulfur. Similarly, in the accession survey, soil-specific phenotypes were observed in seven of the eight accessions.

While each modified soil condition triggered distinct changes in the Col-0 seed ionome compared to standard soil, the steady-state concentration of most elements still appeared to be under relatively tight homeostasis controls. This was indicated by the observation of similar levels of variability for nine elements (Ba, Ca, K, Mg, Mn, Mo, P, S, and Zn) under standard and modified soil conditions (Figure 2). There were however, two notable exceptions: Na in NaCl soil and Li in alkali soil. As the sensitivity of detecting a phenotype in our screen is directly related to the elemental variability in control plants, our ability to detect Na phenotypes under NaCl challenge and Li phenotypes under alkali challenge was greatly diminished. These examples illustrate that, in some cases, the use of modified soil conditions can increase the variability of specific elements and thereby cause phenotypes to be missed that would have otherwise been detected as statistically significant under standard soil conditions.

### Mutant Screen

An extrapolation from our screen of 760 genes supports an estimate that up to 11% (at 95% confidence) of the *A. thaliana* genome might be involved in the regulation of the seed ionome. This estimate is higher than those for *A. thaliana* leaf tissue (2–4%) [7] or for yeast (4.8%) [6]. It is possible that the 11% extrapolated from our results is an over-estimate. For example, 760 genes is still only a small fraction of the genome, and some subgroup(s) of gene functions may be over-represented. However, the seed ionome is expected to be strongly influenced by root and shoot physiological functions as well as sink loading controls within the seed. It is therefore also possible that the seed ionome is dependent on more genes than other plant structures, or a single-cell system like yeast.

While the 760 genes include representatives of all GO annotations, transport-related genes were over-represented by approximately three-fold (Figure S2). Interestingly, this over-representation was actually in contrast to an under-representation of transport-related genes with ionomic phenotypes (Figure S3). This under-representation is similar to that observed in a screen of 4,385 yeast mutants [6]. One possible explanation is that, because of a need to precisely regulate ion concentrations in many cell types, ion homeostasis systems might have evolved with functional redundancies provided by alternative transport pathways [6].

From a subset of genes chosen for further analyses, 15 were found to have reproducible mutant phenotypes (Figure 3). Of these, five were corroborated with two independent alleles and are discussed below. In two cases (ABH1 and CCC), the two independent sets of alleles showed overlapping but non-identical phenotypes, potentially due to different degrees of phenotypic penetrance. Regardless, these five genes provide examples of how ion homeostasis can be influenced by diverse sets of genes, including those involved in regulating gene expression, phospho-regulation, and ion transport. Additional mutations that warrant further consideration are listed in File S1, along with their corresponding ionome profiles and preliminary confidence rankings.

### SOS2: Protein Kinase

An increase in the relative concentration of Na was observed in the seed of *sos2* mutants under all soil conditions tested, including unchallenged (Figure 3). In contrast, evidence from the Purdue Ionomics Information Management System (PiiMS, http://www.ionomicshub.org [32]) (File S2) and a previous study [33] indicate that loss of SOS2 does not alter the leaf ionome under unchallenged conditions. *SOS2* encodes a protein kinase that is thought to be important for a plant’s response to NaCl stress by activating transporters that can regulate the concentration of Na^+^ in the cytosol [34]–[37]. Among the proteins activated by SOS2 are: SOS1, a Na^+^/H^+^ antiporter that effluxes Na^+^ across the PM [38]–[40]; and NHX1, a Na^+^/H^+^ antiporter that sequesters Na^+^ in the vacuole [41]. The activity of the SOS1 Na^+^/H^+^ antiporter has been linked to long-distance Na^+^ transport in plants [42]. The observation that seeds from *sos2* plants over-accumulate Na^+^ relative to Col-0 wild-type is consistent with the involvement of the SOS2 kinase in either reducing the levels of Na^+^ translocated to the seed, or as part of an efflux pathway to exclude Na from accumulating in developing seeds.

### ABH1: Nuclear mRNA Cap Binding Protein

Multiple soil-dependent phenotypes were observed in the seed of *abh1* mutants, with a decrease in S being the only change that was consistent under all growth conditions tested (Figure 3). These changes might be specific to the seed, as no changes have yet been detected in assays of the leaf ionome (File S2). An *abh1* mutant plant was initially characterized as being hypersensitive to ABA [43], [44]. ABH1 encodes the large subunit of the heterodimeric mRNA cap-binding complex (CBC) [43], [44]. The eukaryotic CBC complex binds the 5′ end of RNA polymerase II transcripts and has multiple roles in mRNA biogenesis, including nuclear export [45], 46 and protecting transcripts from nuclease degradation [47]. Loss of ABH1 results in as many as 144 changes in gene expression of two-fold or greater in the leaf, including kinases, transporters and transcription factors (File S3) [48]. ABH1 has also been shown to be involved in alternate splicing [49], miRNA maturation [50]–[53], and possibly protein translation [54]. It is tempting to speculate that the changes in ion homeostasis observed in *abh1* seed are the direct result of altered expression and/or function of one or more genes involved seed in ion homeostasis. However, the possibility cannot be excluded that ionomic changes in the *abh1* seed ionome are the indirect result of changes in whole-plant physiology caused by the ABA hypersensitivity of *abh1* mutants.

### CCC: Cation Chloride Co-transporter

When grown with unmodified soil, *ccc* mutants were observed to produce seed that over-accumulate Ca and S, and under-accumulate Na and K (Figure 3). *CCC* encodes the only known cation chloride co-transporter in *A. thaliana*. Promoter-GUS [55] and public expression profiling analyses [56] suggest that *CCC* is expressed throughout the plant, including roots, shoots, seeds and pollen. In animals, cation chloride co-transporters are divided into three groups based on ion co-transport specificity: K^+^:Cl^-^ (KCC), Na^+^:Cl^-^ (NCC) and Na^+^:K^+^:Cl^-^ (NKCC) [57]. In plants, two lines of evidence suggest a significant role for CCCs in cellular ion homeostasis. First, heterologous expression of CCC from *A. thaliana* in *Xenopus laevis* oocytes provides evidence of Cl^-^ ion transport in the presence of both Na^+^ and K^+^, consistent with transport characteristics observed for the animal NKCC group [55]. Second, gene knockout and RNAi experiments in *A. thaliana* and *Oryza sativa,* respectively, indicate that loss of CCC can disrupt the ability of the plant to regulate the concentrations of Na, K and Cl, especially when grown under salt stress conditions [55], [58]. Both knockout and RNAi silenced plants showed multiple growth defects, consistent with a model in which CCCs play a critical role in ion homeostasis in multiple cell types and tissues. The observation here that a *ccc* mutant over-accumulates Ca and S in seeds raises the question of whether the *in-vivo* ion transport properties of CCC may also include Ca^2+^ or SO_4_^−^, or whether an increase in seed Ca and S could be indirect consequences of pleiotropic changes that affects ion loading or fluxes within the transpiration stream or nutritional loading into seeds.

### At3g14280: Protein with Obscure Features

A decrease in seed S was observed in mutant plants with T-DNA insertions in the gene At3g14280 under all growth conditions tested (Figure 3). The protein encoded by gene At3g14280 is currently annotated as a protein with obscure features (POF) [59], [60], with putative orthologs in monocots, dicots and mosses; none of which are annotated with known motifs, domains, or biological functions. The protein encoded by At3g14280 is predicted to be soluble [61], and public expression profiling data suggests it is expressed throughout the plant, including roots, shoots and pollen [56]. This highlights the potential of using a seed ionome screen to provide experimental evidence for annotating unknown genes with potential physiological functions. From our first pass screen of 760 genes, 45 “unknowns” were scored with potential phenotypes worthy of follow up analyses (File S1). In the case of the POF/unknown encoded by At3g14280, we suggest its designation as a sulfur homeostasis gene (SHO), as two independent T-DNA insertions in its coding sequence result in a lower concentration of S in the *A. thaliana* seed.

### CNGC2: Cyclic Nucleotide Gated Channel

An increase in Ca and a decrease in Mg were observed in the seed of *cngc2* mutants under NaCl challenge conditions (Figure 3). Whether these changes might be specific to the seed is not yet clear, but current evidence from the PiiMS database suggests that the leaf ionome is unaltered under an unchallenged soil condition (File S2). Cyclic nucleotide gated channels (GNGCs) are nonselective cation-conducting channels that can be activated by cyclic nucleotides. 20 CNGC isoforms (CNGC 1–20) have been identified in *A. thaliana* [62]. Electrophysiological experiments in *Xenopus laevis* oocytes, human embryonic kidney (HEK) cells and *A. thaliana* guard cells suggest that CNGC2 is permeable to cations including K^+^ and Ca^2+^, [63]–[65]. Loss of CNGC2 results in reduced plant health with a specific hypersensitivity to high Ca^2+^ levels (10 mM) [66], [67]. While the possibility cannot be excluded that the changes in ion homeostasis for *cngc2* are an indirect consequence of reduced plant health, other CNGC isoforms have been implicated in the maintenance of ion homeostasis. For example, *cngc3* mutant seedlings under-accumulate K when grown on high levels of NaCl or KCl [68], *cngc1* mutant seedlings under-accumulate Ca [69], and antisense plants under-expressing CNGC10 under-accumulate Ca, Mg and K in shoot tissue [70]. Evidence from studies on CNGC18 are consistent with the potential of CNGCs to change the accumulation of Ca [71]. These examples of *cngc* mutants that under-accumulate Ca are in contrast to our finding that Ca is over-accumulated in *cngc2* seed. The observation that *cngc2* mutants also under-accumulate Mg in seeds is consistent with previous reports of a strong correlation between Ca and Mg concentrations [2], and raises the question of how Mg^2+^ transport might be influenced by CNGC2.

### Accession Screen

Changes in the seed ionome, relative to Col-0, were observed for eight of the nine accessions studied. This is similar to what was seen for a seed and leaf ionome comparison in a previous study with 12 accessions grown with soil amended with subtoxic levels of various elements, including Cd and As [14]. However, our results using plants grown on four different soils indicate that most of the differences are likely to be soil-dependent.

Only one of the nine ionomic changes observed in accessions screened under all four soil conditions occurred consistently under all soil conditions. Nevertheless, the observed high frequency of natural variation is consistent with the hypothesis that the seed and leaf ionomes are both sensitive indicators for changes in genetic networks that accompany adaptive changes in the evolution of plant accessions [72]. In the context of the leaf ionome, natural variation has already been used to identify several genes of ionomic importance, for example: *MOT1*, Mo concentration [19]; *FPN2*, Co concentration [22]; *APR2*, sulfate concentration [20]; and *HKT1*, Na concentration [18], [21], [25], [73].

Importantly, two accessions analyzed here show increased seed concentrations for Zn (Mrk-0) or Fe (Kas-1). As these two elements are considered important nutrients for biofortification of seeds, understanding the genetic basis of this natural variation might be instructive towards genetic improvements in crop plants. In the case of increased Zn in Mrk-0 seed, this change was only seen in plants grown under NaCl and alkali challenges. This suggests that understanding a NaCl or alkali stress response in Mrk-0 might provide insights into genetic networks that regulate Zn homeostasis.

In the case of increased Fe in Kas-1 seed, this change was seen for both untreated and alkali soil conditions. A seed analysis was not possible for the NaCl or heavy metal challenges, as these plants were highly sensitive to the NaCl and heavy metal conditions used here, and produced very few seed. However, a previous study with a different amended soil treatment also showed an increase in seed Fe [14]. Interestingly, the previous study did not see any Fe changes in the leaf ionome. This suggests that the increased levels of Fe in Kas-1 seeds are not soil-dependent, and that the mechanism responsible has a seed specific feature.

### Seed and Leaf Ionomes are Distinct

Previous studies have shown that the relative elemental compositions of root, leaf and seed tissues are very different [14], [16], [17], [74]. For nine of the 15 seed ionome mutants identified here, an initial comparison with changes in the leaf ionome was possible through information available either from a public database (PiiMS) or the literature. With this subset of mutants, there were no examples in which elemental concentrations were altered in the same way (File S2). Similar results have emerged from analyses of three RIL populations of *A. thaliana* [17]. These examples support an expectation that the regulatory controls over the seed ionome are distinct from those that regulate the leaf, despite a dependence of the seed on vegetative tissues for their source of nutrients. Thus, in working towards a goal of biofortifying seeds with increased levels of mineral nutrients, specific attention will be required to understand the ion homeostasis controls that are specific to the seed ionome.

### Conclusions

Here, we identified seed ionome functions for 15 genes, five of which were corroborated with two independent mutant alleles. Significantly, all 15 mutants showed at least one ionomic change that could be identified in plants grown under standard conditions. This suggests that the sole use of a standard soil condition might provide the most efficient strategy for a first pass screen of the entire genome to identify genes that can impact the seed ionome. Nevertheless, our results show that modified soil conditions have significant influence on the seed ionome (Figure 1), and can be used to uncover soil-dependent ionomic phenotypes in mutants (Figure 3) and naturally occurring accessions (Figure 4). Thus, a comprehensive understanding of the genetic networks governing ion homeostasis will eventually require careful attention to factors impacting the soil environment.

## Materials and Methods

### Resources

Seeds for the accessions [75], the SALK [76], SAIL [77] and WiscDsLox [78] T-DNA disruption lines and the CSHL [79] transposon disruption lines were obtained from the Arabidopsis Biological Resource Facility at Ohio State University. Seeds for SM [80] transposon disruption lines were obtained from the NASC European Arabidopsis Stock Centre. The locations of all inserts were estimated using flanking sequences available through the Salk SIGnAL T-DNA Express portal (http://signal.salk.edu/cgi-bin/tdnaexpress). Plant lines in the Wisconsin Collection [81] were isolated by individual labs from pooled seed and are available from the Harper Lab. Seeds designated as being from the Harper Lab Collection and all multiple knockout plant lines are also available from the Harper Lab. Seed lines with the prefix “FN” carry fast neutron deletions [7] and are available from the Salt Lab. The *sos2-1* allele [37] carries a fast neutron deletion and is available from the Arabidopsis Biological Resource Facility at Ohio State University.

### Untreated Soil Condition

Seeds were sown on plates containing ½ strength Murashige and Skoog basal salts, 0.05% MES (pH 5.7) and 1% agar. The seeds were stratified (4°C, dark) for 48 h and then grown at room temperature under 24 h light for 10 d. Seedlings were transplanted to soil (Sunshine LB-2 or Sunshine SMB283) and grown in a growth chamber at 21°C under long-day light conditions (16 h light/8 h dark) until bolting. After bolting, the plants were moved to a greenhouse where they were allowed to grow to maturity. The plants were frequently subirrigated by soaking the flats for ∼16 h in an ebb-and-flow tray with an excess of 1/10 strength Hoaglands #2 basal salts supplemented with 5 µM Sprint138 Iron Chelate (Becker-Underwood, Ames, IA, USA).

### 75 mM NaCl Soil Condition

Plants were grown as described for the untreated soil condition until bolting. Upon bolting, plants were moved to a greenhouse and subirrigated by soaking the flats for ∼16 h in an ebb-and-flow tray with an excess of 1/10 strength Hoaglands #2 supplemented with 5 µM Sprint138 Iron Chelate (Becker-Underwood, Ames, IA, USA) and 75 mM NaCl. This watering strategy was used to maintain a constant 75 mM NaCl challenge to the plants over the course of the experiment. Unless the NaCl concentration in the soil was allowed to equilibrate with an excess of fertilizer +75 mM NaCl, NaCl would accumulate in the soil resulting in a NaCl challenge far greater than 75 mM.

### Heavy Metal Soil Condition

Seeds were sown on plates containing ¼ strength Murashige and Skoog with Gamborg’s vitamins, 10 mM MES (pH 5.6) and 1% phytoagar, and two week old seedlings were transplanted to soil. Heavy metal-containing soil was made by mixing 1 kg of soil (Sunshine Basic Mix 2) with 1 L heavy metal solution [0.005% AlCl_3_, 0.005% KH_2_AsO_4_, 0.005% CdCl_2_, 0.01% CoCl_2_ and 0.0025% each of BaCl_2_, CsNO_3_, K_2_CrO_4_, CuSO_4_, Ca(NO_3_)_2_, Fe(SO_4_)_3_, MnCl_2_, MgCl_2_, LiCl, RbCl and ZnSO_4_ (w/v)], and the soil was split evenly among three trays. Trays were subirrigated thrice weekly with 700 ml of ¼ strength Hoagland’s #2 basal salts supplemented with 25 µg/L Sprint 330 Iron Chelate (Becker-Underwood, Ames, IA, USA).

### Alkali Soil Condition

Seedlings were grown on plates containing Gamborg’s B5 media until they reached the 3–4 true leaf stage and were transplanted to high pH soil. High pH soil was prepared by supplementing Sunshine LB-2 soil with CaO (7.8g CaO/kg dry soil). The plants were subirrigated frequently with ¼ strength Hoagland’s #2 basal salts supplemented with 5 µM Sprint138 Iron Chelate (Becker-Underwood, Ames, IA, USA).

### Elemental Concentration Measurements

Elemental analysis was performed using a Varian Vista PRO ICP-AES after a HNO_3_ digestion. Samples were placed in 15 ml Falcon polypropylene centrifuge tubes (Fisher Scientific, Waltham, MA, USA) to which 0.5–1 ml of trace metal grade HNO_3_ was added. The samples tubes were capped and allowed to react overnight followed by heating in an oven to 80°C for 2 h. After cooling, tubes were brought up to a volume of 3.5 ml in the case of a 0.5 ml HNO_3_ digestion or 5 ml in the case of a 1 ml HNO_3_ digestion. Analysis was conducted using conventional ICP-AES methods with the addition of a 0.5 ml Y +10 g CsNO_3_/L solution introduced into the sample uptake for internal standardization.

### Calculations

Sample weights were calculated using a method derived from that of Lahner et al. [7]. Briefly, sample weights were calculated by comparing the concentration (signal) of specific elements in experimental samples to weighed control samples. These calculations are based on the assumption that the signal/weight ratio (albeit different for every element) is consistent for experimental and control samples. In contrast to Lahner et al., [7], the subset of elements used in the weight calculations was not fixed, but rather elements were included if the variation in signal/weight ratio was 25%RSD or less. The concentrations of elements within mutant plants relative to the controls were considered in terms of z-Score (number of standard deviations from control mean) using:

Equation 1:
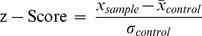
and in terms of percent change using:

Equation 2:
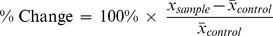
Where 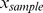 is an individual sample, 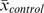 is the mean of control samples and 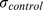 is the standard deviation of the control samples. Data analysis was done in the Harper Lab using the PERL and R programming languages.

## Supporting Information

Figure S1 **75 mM NaCl soil modification does not alter Col-0 seed size.** Average results (±SE) of four independent experiments are presented for untreated (open bars, n = 12 plants) and 75 mM NaCl (crosshatched bars, n = 12 plants) soil conditions. Seed sizes were measured from scanned images as two-dimensional areas using the ImageJ software package. No statistically significant differences were observed between untreated and 75 mM NaCl soil conditions (p = 0.7605, Welch’s t-test).
(PDF)

Figure S2 **Gene ontology analyses comparing the genes from the mutant screen to the** ***A. thaliana*** **genome.** Gene Ontology (GO) analyses were done using gene ontology annotations from TAIR (http://www.arabidopsis.org). Three aspects of each gene product are considered individually: biological process, cellular component and molecular function. Each aspect is divided up into non-overlapping functional categories. Columns represent the percentage of genes within a functional category. The most significant difference between the genes analyzed in this study and the *A. thaliana* genome is an approximately three-fold increase in the number of transport-related genes in the Biological Process and Molecular Function categories. Eight of the 760 genes screened were not included in the GO analysis because they were represented by fast-neutron mutant lines and their identity is unknown.
(PDF)

Figure S3 **Gene ontology analyses comparing genes with potential phenotypes to genes without potential phenotypes.** Analyses were done as described in Figure S2. Genes were considered to have a potential ionomic phenotype if they were identified as candidates in the first pass screen. Eight of the 760 genes screened were not included in the GO analysis because they were represented by fast-neutron mutant lines and their identity is unknown. Transport-related genes from both the Biological Process and Molecular Function GO categories were slightly less likely to show a putative phenotype. This suggests that their approximately three-fold over-representation in the mutant screen (Figure S2) did not produce a disproportionate increase in the estimate that 7–11% of the *A. thaliana* genome is involved in regulating seed ion homeostasis.
(PDF)

File S1 **Cumulative results of trace element screen.** The concentrations of elements in experimental plants relative to the Col-0 controls are represented in terms of z-Scores (number of standard deviations from the control mean). Data represents the median z-Scores of an experimental line within an individual flat. Boxes shaded in gray represent circumstances for which no data was collected. A change in elemental concentration is considered statistically significant if different from the control mean by three or more standard deviations (z-Score ≥3 or z-Score ≤ −3). Statistically significant changes in elemental concentrations are represented by blue (lower concentration) and yellow (higher concentration) shaded boxes. The phenotype of a line is described for each growth condition in one of four possible ways: reproducible, low confidence, inconsistent and none. A phenotype is considered “reproducible” if consistent within multiple trays of the same soil condition, across different soil conditions, or for independent alleles of the same gene. A phenotype is considered “low confidence” if observed in the first pass screen and not chosen for follow up analysis. A phenotype is considered “inconsistent” if observed in the first pass screen and not corroborated by a follow up analysis. A phenotype description of “none” indicates that no statistically significant changes in elemental concentrations were observed. IG#’s represent internal seed stock designations. Except in the case of double knockouts, common names and TAIR descriptions were obtained from the TAIR website (http://www.arabidopsis.org) and were current on 6/1/12.
(XLS)

File S2 **Elemental abundances in vegetative tissue.** The elemental contents of mutant plants relative to the tray average is represented in terms of z-Scores (number of standard deviations from the tray mean). Data represents the median z-Scores of an experimental line within an individual flat. Boxes shaded in gray represent circumstances for which no data was collected. A change in elemental accumulation is considered statistically significant if different from the tray average by three or more standard deviations (z-Score ≥3 or z-Score ≤ −3). Statistically significant over- and under-accumulations are represented by yellow and blue shaded boxes respectively. IG#’s represent internal seed stock designations. Data was obtained from the PiiMS database.
(XLS)

File S3 **Gene expression is altered in** ***abh1*** **mutant plants.** Data represents the smallest fold change in gene expression between *abh1* and wild-type from two biological replicates. Microarray data used for this analysis is publicly available in the NCBI GEO database (http://www.ncbi.nlm.nih.gov/projects/geo/, GSE7112) and was originally published by Kuhn et al., 2008 [48].
(XLS)
