# Supplementary material for: Elemental Concentrations in the Seed of Mutants and Natural Variants of Arabidopsis thaliana Grown under Varying Soil Conditions
Source: PLoS One. 2013 May 6;8(5):e63014. doi: 10.1371/journal.pone.0063014 (PMC3646034; doi:10.1371/journal.pone.0063014)
Supplement: Figure S1 — 75 mM NaCl soil modification does not alter Col-0 seed size. Average results (±SE) of four independent experiments are presented for untreated (open bars, n = 12 plants) and 75 mM NaCl (crosshatched bars, n = 12 plants) soil conditions. Seed sizes were measured from scanned images as two-dimensional areas using the ImageJ software package. No statistically significant differences were observed between untreated and 75 mM NaCl soil conditions (p = 0.7605, Welch’s t-test). (PDF) [file pone.0063014.s001.pdf]

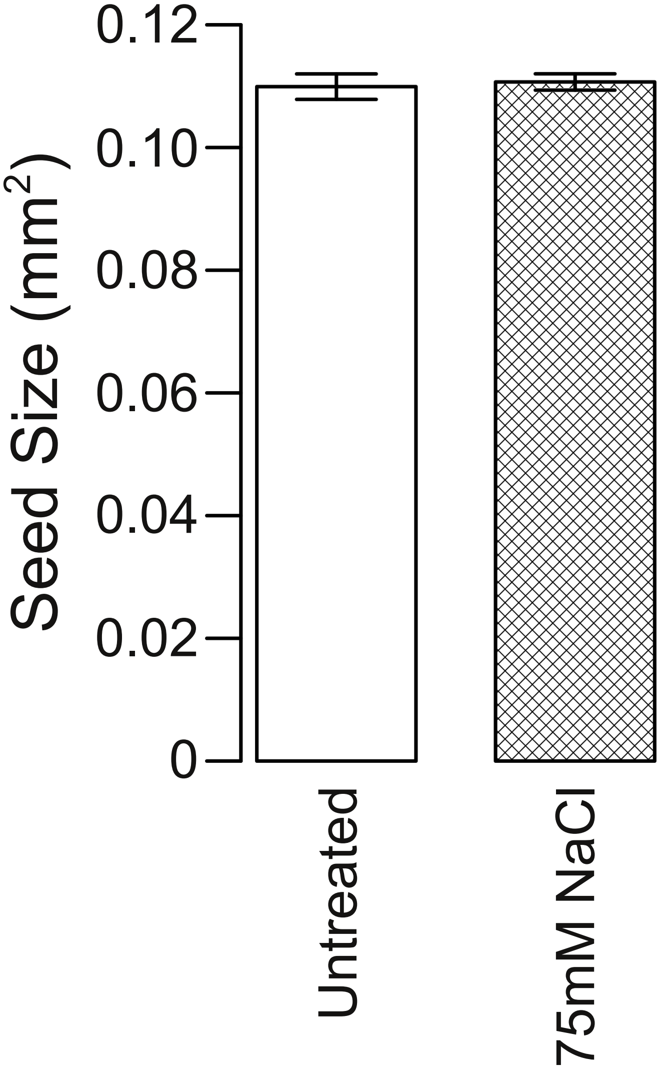

Figure S1. 75 mM NaCl soil modification does not alter Col-0 seed size. Average results (±SE) of four independent experiments are presented for untreated (open bars, n = 12 plants) and 75 mM NaCl (crosshatched bars, n = 12 plants) soil conditions. Seed sizes were measured from scanned images as two-dimensional areas using the ImageJ software package. No statistically significant differences were observed between untreated and 75 mM NaCl soil conditions (p = 0.7605, Welch's t-test).
