# Supplementary material for: Elemental Concentrations in the Seed of Mutants and Natural Variants of Arabidopsis thaliana Grown under Varying Soil Conditions
Source: PLoS One. 2013 May 6;8(5):e63014. doi: 10.1371/journal.pone.0063014 (PMC3646034; doi:10.1371/journal.pone.0063014)
Supplement: Figure S2 — Gene ontology analyses comparing the genes from the mutant screen to the A. thaliana genome. Gene Ontology (GO) analyses were done using gene ontology annotations from TAIR (http://www.arabidopsis.org). Three aspects of each gene product are considered individually: biological process, cellular component and molecular function. Each aspect is divided up into non-overlapping functional categories. Columns represent the percentage of genes within a functional category. The most significant difference between the genes analyzed in this study and the A. thaliana genome is an approximately three-fold increase in the number of transport-related genes in the Biological Process and Molecular Function categories. Eight of the 760 genes screened were not included in the GO analysis because they were represented by fast-neutron mutant lines and their identity is unknown. (PDF) [file pone.0063014.s002.pdf]

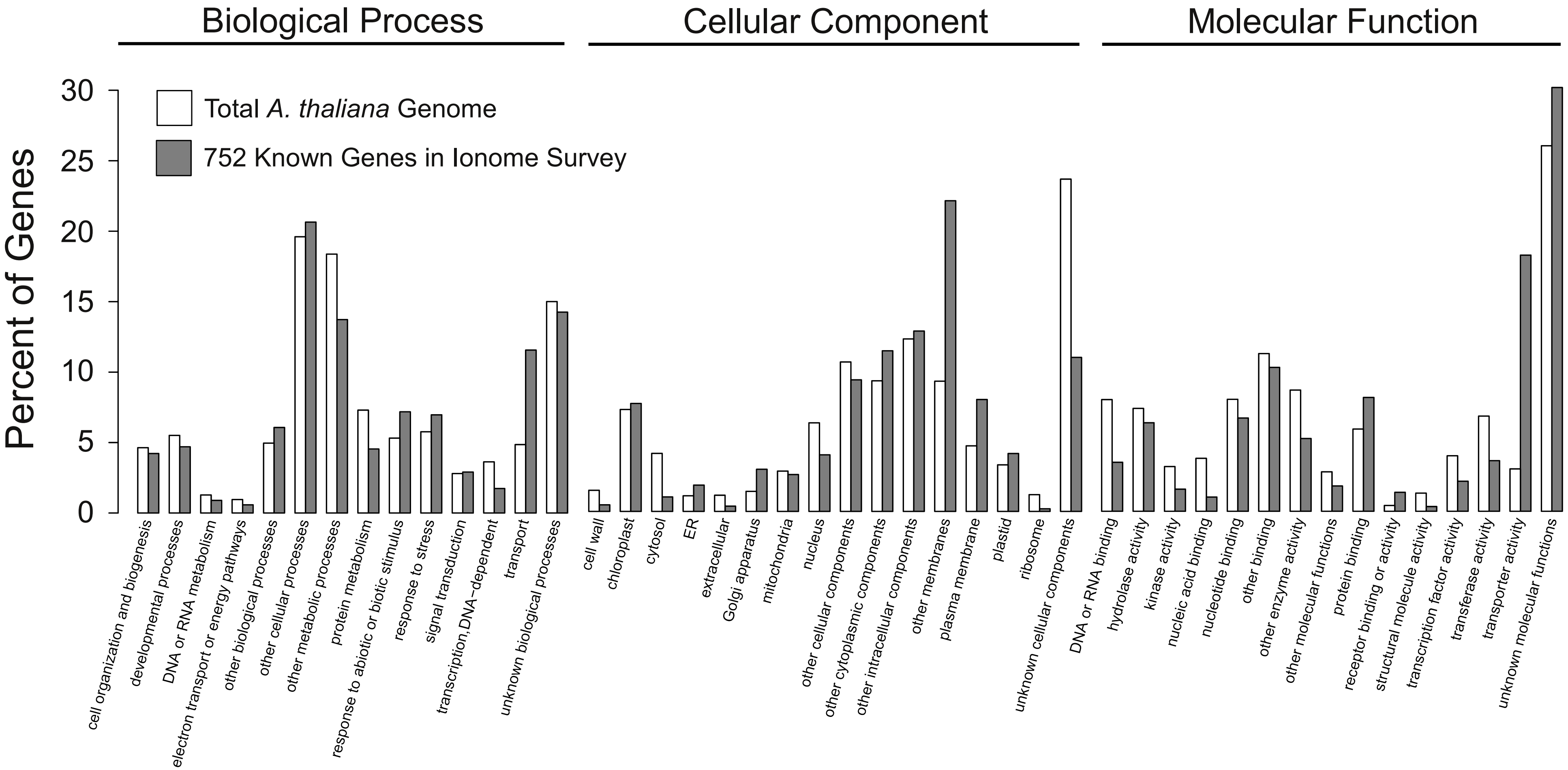

Figure S2. Gene ontology analyses comparing the genes from the mutant screen to the *A. thaliana* genome. Gene Ontology (GO) analyses were done using gene ontology annotations from TAIR (http://www.arabidopsis.org). Three aspects of each gene product are considered individually: biological process, cellular component and molecular function. Each aspect is divided up into non-overlapping functional categories. Columns represent the percentage of genes within a functional category. The most significant difference between the genes analyzed in this study and the *A. thaliana* genome is an approximately three-fold increase in the number of transport-related genes in the Biological Process and Molecular Function categories. Eight of the 760 genes screened were not included in the GO analysis because they were represented by fast-neutron mutant lines and their identity is unknown.
