# Supplementary material for: Elemental Concentrations in the Seed of Mutants and Natural Variants of Arabidopsis thaliana Grown under Varying Soil Conditions
Source: PLoS One. 2013 May 6;8(5):e63014. doi: 10.1371/journal.pone.0063014 (PMC3646034; doi:10.1371/journal.pone.0063014)
Supplement: Figure S3 — Gene ontology analyses comparing genes with potential phenotypes to genes without potential phenotypes. Analyses were done as described in Figure S2. Genes were considered to have a potential ionomic phenotype if they were identified as candidates in the first pass screen. Eight of the 760 genes screened were not included in the GO analysis because they were represented by fast-neutron mutant lines and their identity is unknown. Transport-related genes from both the Biological Process and Molecular Function GO categories were slightly less likely to show a putative phenotype. This suggests that their approximately three-fold over-representation in the mutant screen (Figure S2) did not produce a disproportionate increase in the estimate that 7–11% of the A. thaliana genome is involved in regulating seed ion homeostasis. (PDF) [file pone.0063014.s003.pdf]

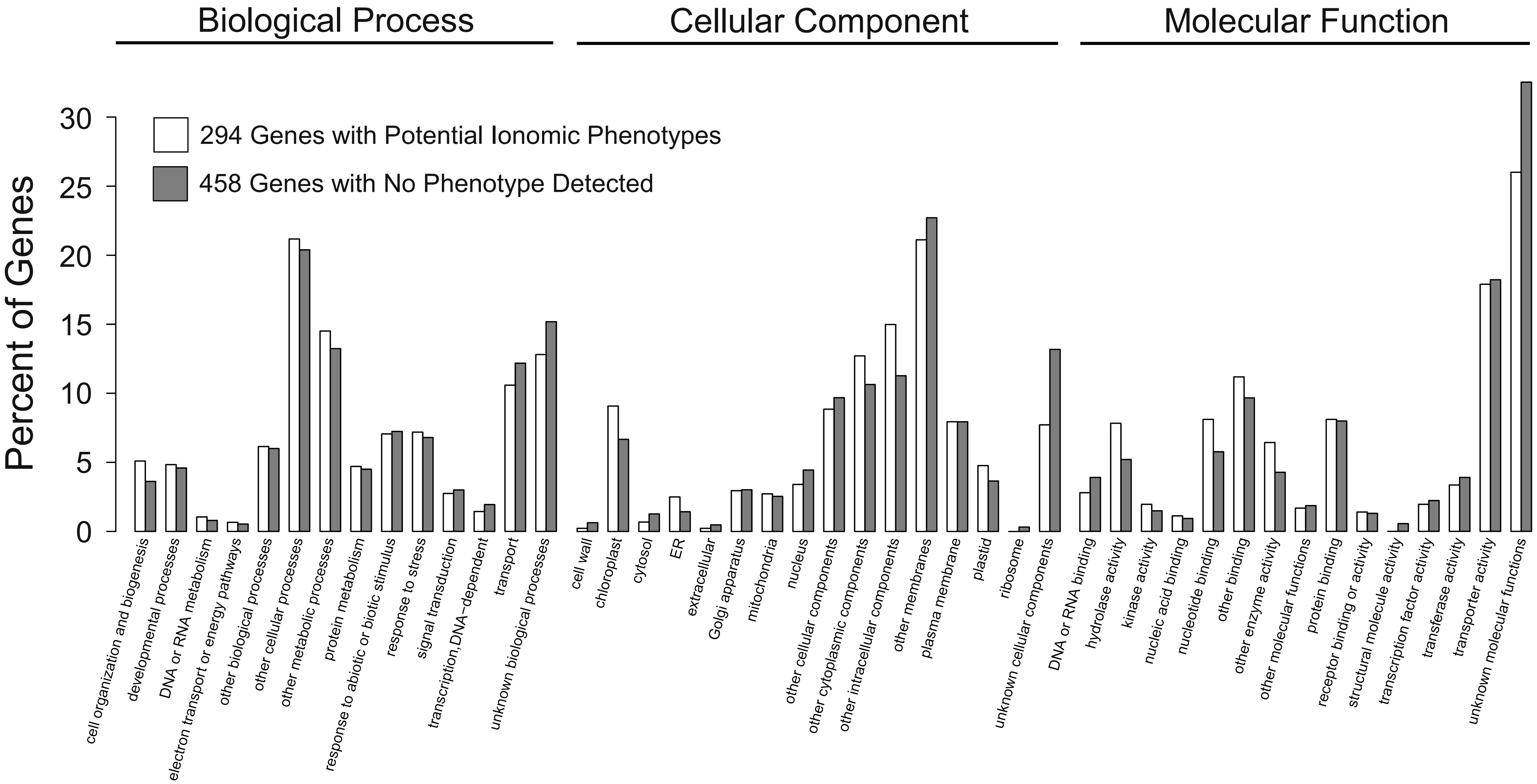

Figure S3. Gene ontology analyses comparing genes with potential phenotypes to genes without potential phenotypes. Analyses were done as described in Figure S2. Genes were considered to have a potential ionomic phenotype if they were identified as candidates in the first pass screen. Eight of the 760 genes screened were not included in the GO analysis because they were represented by fast-neutron mutant lines and their identity is unknown. Transport-related genes from both the Biological Process and Molecular Function GO categories were slightly less likely to show a putative phenotype. This suggests that their approximately three-fold over-representation in the mutant screen (Figure S2) did not produce a disproportionate increase in the estimate that 7-11% of the *A. thaliana* genome is involved in regulating seed ion homeostasis.
